# Potentials and challenges of diffusion-weighted magnetic resonance imaging in radiotherapy

**DOI:** 10.1016/j.ctro.2018.09.002

**Published:** 2018-09-20

**Authors:** Sara Leibfarth, René M. Winter, Heidi Lyng, Daniel Zips, Daniela Thorwarth

**Affiliations:** a Section for Biomedical Physics, Department of Radiation Oncology, University Hospital Tübingen, Germany; b Department of Radiation Oncology, University Hospital Tübingen, Germany; c Department of Radiation Biology, Norwegian Radium Hospital, Oslo University Hospital, Norway

## Abstract

**Purpose:**

To review the potential and challenges of integrating diffusion weighted magnetic resonance imaging (DWI) into radiotherapy (RT).

**Content:**

Details related to image acquisition of DWI for RT purposes are discussed, along with the challenges with respect to geometric accuracy and the robustness of quantitative parameter extraction. An overview of diffusion- and perfusion-related parameters derived from mono- and bi-exponential models is provided, and their role as potential RT biomarkers is discussed. Recent studies demonstrating potential of DWI in different tumor sites such as the head and neck, rectum, cervix, prostate, and brain, are reviewed in detail.

**Conclusion:**

DWI has shown promise for RT outcome prediction, response assessment, as well as for tumor delineation and characterization in several cancer types. Geometric and quantification robustness is challenging and has to be addressed adequately. Evaluation in larger clinical trials with well designed imaging protocol and advanced analysis models is needed to develop the optimal strategy for integrating DWI in RT.

## Highlights

• Discussion of DW imaging protocols and imaging setup.
• Discussion of mono- and bi-exponential models for quantitative parameter extraction.
• Review of recent publications investigating potential benefits of using DWI in RT, including detailed synoptic table.
• Detailed discussion of geometric and quantitative accuracy of DW imaging and DW-derived parameters.

## 1 Introduction

The integration of magnetic resonance (MR) imaging into radiotherapy (RT) represents an active field of ongoing research. Anatomical MR imaging may be highly beneficial for the precise delineation of the gross tumor volume (GTV) [1], [2]. Moreover, functional imaging might allow for biological and physiological tumor characterization. It might therefore be a basis for treatment individualization strategies such as dose painting [3], [4], [5], as well as a tool for treatment monitoring and early response assessment [6], [7], [8]. In addition to standalone MR scanners and combined positron emission tomography (PET)/MRI, first commercial MR-linac systems are available and being implemented at clinical sites [9], [10], [11].

One of the most promising functional MR imaging methods for RT applications is diffusion weighted imaging (DWI). This paper provides a review of current research about using DWI for RT purposes. It addresses the derivation of biomarkers from DWI, potential benefits of using DWI-derived biomarkers for RT adaptation and response assessment, as well as the specific challenges with respect to integrating DWI into RT.

## 2 Basic physics of DWI

DWI can be achieved by placing two additional diffusion sensitizing gradients on each side of the 180° radio frequency (RF) pulse of a spin-echo sequence, as introduced by Stejskal and Tanner [12]. The magnitude of diffusion weighting can then be expressed by the *b*-value, which is defined as

$$b=\gamma^{2}G^{2}\delta^{2}(\Delta -\delta /3),$$

where γ is the gyromagnetic ratio, *G* is the magnitude of the diffusion sensitizing gradients, δ is the temporal duration of each of the gradients, and Δ is the time interval between the application of the gradients.

The diffusion sensitizing gradients do not have an effect on stationary spins, since any phase accumulation from the first gradient lobe is reversed by the second. However, for non-stationary spins a non-vanishing phase shift remains, with the magnitude of the shift being determined by the respective trajectory performed between the start of the first and the end of the second gradient. This results in a loss in signal of an ensemble of diffusing spins. A higher *b*-value chosen for diffusion-weighing results in a more pronounced signal loss. Diffusion weighting thus provides an additional contrast mechanism for MR image acquisition. When acquiring several images with different *b*-values, quantitative diffusion- and perfusion-related parameters can be derived by applying a model describing the *b*-value dependent signal loss.

The microstructural organization in tissue, and consequently the local diffusion coefficients, are in general anisotropic. In anatomical regions with a strong anisotropy, such as the brain, the directional information can be highly relevant and might be obtained by diffusion tensor imaging (DTI). In DTI, directional information is obtained by applying diffusion-weighted gradients in at least six directions and deriving a diffusion tensor [13]. For many applications in RT, however, directional information is not required. Measurements with diffusion weighting in the three orthogonal spatial directions can then be combined into a single directionally averaged diffusion weighted image. As diffusion coefficients derived from these averaged images are identical to the trace of the diffusion tensor divided by a factor of three, corresponding images are also referred to as *trace images* [14].

## 3 DWI models and potential biomarkers

Studies have revealed that signal loss at low *b*-values is dominated by perfusion effects, whereas signal loss at high *b*-values is dominated by diffusion [15]. The *b*-value representing the transition between the perfusion and diffusion effects depends on the vascular properties of the tissue. As for head and neck, transition *b*-values as different as 100 $s/{\mathrm{mm}}^{2}$ [16] and 300 $s/{\mathrm{mm}}^{2}$ [17] were proposed in literature without systematic site-specific derivation. A more quantitative approach to possibly determine the transition between perfusion and diffusion regimes has been provided in the context of non-small cell lung cancer [18].

Different models can be applied to extract quantitative parameters from the images, which might be potential biomarkers for RT. A more advanced model might describe the *b*-value dependent signal loss more accurately than a simpler one, and provide more insights into tissue organization. However, it may perform worse if the quality of the evaluated data is not sufficient. It is thus crucial to take robustness of the evaluation into account.

### 3.1 Mono-exponential model

The simplest and most commonly used model for analysis of DW images assumes a mono-exponential decay of the signal *S* with increasing *b*-values

(1) $$S=S_{0}\exp(-\mathrm{ADC}\cdot b)\text{.}$$

The fit parameter in the exponent is called *apparent diffusion coefficient* (ADC). When derived from the high *b*-value range, it describes the effective water diffusion in the tissue [15]. In contrast, coefficients derived from the low *b*-value range predominantly contain perfusion information, whereas, when derived from a mixture of low and high *b*-values, both perfusion and diffusion effects are included. The biological meaning of the ADC value therefore strongly depends on the *b*-values included in the analysis, making it difficult to compare results across studies when based on different *b*-values. The mono-exponential model is, in contrast to other models, generally implemented in the vendor-provided scanner software. Compared to other models, it also has the lowest requirements for image acquisition in terms of the number of *b*-values and measurement accuracy. This is especially beneficial if diffusion should be analyzed on a voxel level.

### 3.2 IVIM model

By modeling signal decay using a bi-exponential function, perfusion and diffusion parameters can be taken into account separately

(2) $$S=S_{0}\left(f\exp(-D^{*}\cdot b)+\left(1-f\right)\exp(-D\cdot b)\right)\text{.}$$

**Fig. 1:**
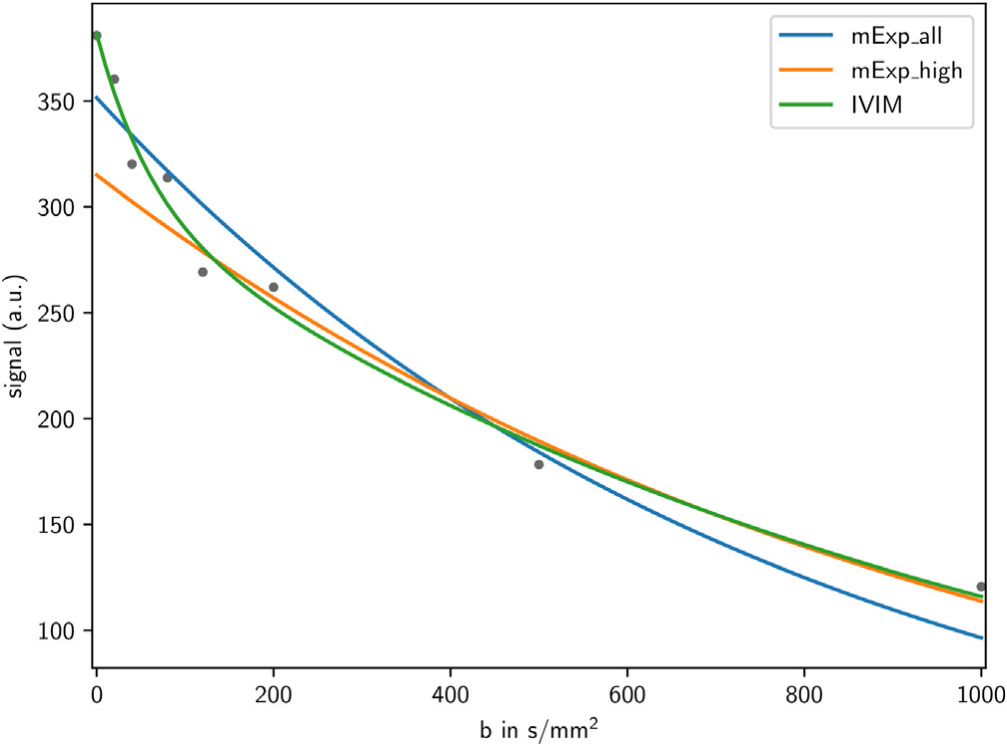
Applying different models to extract quantitative parameters from the mean DWI signals derived within the gross tumor volume (GTV) of a oropharyngeal cancer patient. *Blue*: mono-exponential model (mExp) using high and low *b*-values (mExp_all), *orange*: mExp using only high *b*-values >200 $s/{\mathrm{mm}}^{2}$ (mExp_high), *green*: intra-voxel incoherent motion (IVIM) model. Fit parameters are ADC = 1293 × $10^{-6}\;{\mathrm{mm}}^{2}/s$ (mExp_all), $\text{ADC}=1018\times 10^{-6}\;{\mathrm{mm}}^{2}/s$ (mExp_high), and $f=0.21,D=958\times 10^{-6}\;{\mathrm{mm}}^{2}/s$, $D^{*}=16,500\times 10^{-6}\;{\mathrm{mm}}^{2}/s$ (IVIM). (For interpretation of the references to colour in this figure legend, the reader is referred to the web version of this article.)

This model is called *intravoxel incoherent motion* (IVIM). Perfusion fraction *f* and pseudo-diffusion coefficient $D^{*}$ are derived as perfusion-related parameters, whereas true diffusion is quantified by the diffusion coefficient *D*. Fig. 1 shows a comparison of applying IVIM and the mono-exponential model in an exemplary case of DWI acquired in a head and neck cancer patient. Generally, the IVIM can reproduce the DWI-related signal decay more accurately. However, it requires higher data quality as it contains four fit parameters instead of two in mono-exponential modeling. Also, the model may be over-parametrised, and especially model parameter $D^{*}$ might be not robustly estimated [19], [20]. For the application of IVIM, ten or more *b*-values are considered reasonable, including multiple *b*-values in the low range to capture perfusion effects. The IVIM model is usually only applied within region-based evaluations [19], [21], likely due to the lack of robustness in voxel-by-voxel analysis.

An approximation of the original IVIM model is provided by the so-called *simplified IVIM* model [22], [23], [24]. As in tissue the condition $D^{*}\gg D$ is usually met, the first term of Eq. (2) can be neglected for high *b*-values. Thus, Eq. (2) can be approximated by the mono-exponential relation

(3) $$S=S_{0}(1-f)\exp(-D\cdot b)\text{.}$$

By applying the substitution

(4) $$S_{0}(1-f)\equiv S_{\mathrm{int}},$$

we can write

(5) $$S=S_{\mathrm{int}}\exp(-D\cdot b)\text{.}$$

$S_{\mathrm{int}}$ can be determined by a mono-exponential fit applied to the high *b*-value range (as in the mono-exponential model in Eq. (1)). According to Eq. (4) the perfusion fraction *f* of the IVIM model can then be derived as

(6) $$f=1-S_{\mathrm{int}}/S_{0},$$

where $S_{0}$ can be directly obtained by measurement without diffusion gradient (b=0). An advantage of the simplified over the original IVIM model is the reduced requirements in the number of *b*-values, as the steep initial perfusion-related signal loss does not have to be captured by measurements.

### 3.3 Kurtosis model

Both the mono-exponential model and the IVIM model assume an isotropic Gaussian diffusion. However, due to the interaction of water molecules with microstructural components such as cell membranes, there will be a deviation from pure Gaussian diffusion. A correction to the models above is to take account for this non-Gaussian diffusion behaviour by an additional fit parameter, the kurtosis coefficient *K*, which is derived along with the kurtosis-corrected diffusion coefficient $D_{K}$ [25], [26]. *K* might contain information about the microstructural organization of the tissue which is complementary to the perfusion- and diffusion-based parameters contained in the mono-exponential and the IVIM model. When adding the kurtosis correction, the mono-exponential model (cf. Eq. (1)) changes to

(7) $$S=S_{0}\exp\left(-b\;D_{K}+\frac{1}{6}\;b^{2}\;D_{K}^{2}\;K\right)\text{.}$$

One pre-requisite for using this model is the acquisition of very high *b*-values, which should exceed 1000 $s/{\mathrm{mm}}^{2}$ [25]. There is only a limited number of studies applying this model for RT applications so far, but some promising results have been published recently [26], [27], [28].

### 3.4 Interpretation of DWI parameters and relation to other biomarkers

The detailed interpretation of parameters derived from DWI with respect to underlying biological-physiological conditions is not straightforward and still a matter of debate. Diffusion-related parameters, such as the ADC derived from high *b*-value images, and the diffusion parameter *D* from the IVIM model, are usually related to tissue cellular density, extracellular-space tortuosity, and the integrity of cellular membranes [29]. As an example, low ADC has been associated with high cellularity in histological sections in various cancer types [15]. During the course of RT diffusion tends to increase due to cell membrane disruption and treatment-induced cell death. Especially high diffusivity within the tumor is observed in necrotic, as well as in inflammated regions [29], [30].

Low ADC has also been found to be related to other biological parameters such as a high [^18^F]-fluorodeoxyglucose (FDG) uptake in PET images [31] and a high expression of the Ki67 proliferation marker [32], [33]. The perfusion parameter *f* derived from IVIM has been less studied. Positive correlations between *f* and vascular density [34], [35] as well as perfusion parameters derived from dynamic contrast enhanced (DCE) MR images [36], [37] have been shown, supporting utilization of *f* parametric maps as a biomarker of tumor vascularization and blood flow.

## 4 DWI acquisition details

### 4.1 Choice of *b*-values

The imaging protocol for DWI should be carefully designed. A crucial point to consider is the choice of *b*-values. The actual choice should depend on the context in which the images are acquired and on the model chosen for analysis. Given the model used for DWI data analysis (cf. Section 3) provides an adequate description of the signal decay, a higher number of *b*-values generally lead to a better estimation of fit parameters as well as a better quantification of fit accuracy. For advanced models considering both perfusion and diffusion information, various *b*-values in the low as well as in the high *b*-value range are necessary. However, since the signal decreases with higher *b*-value, more averages should be performed for high *b*-value acquisitions to achieve an acceptable signal to noise ratio. For a voxel-by-voxel analysis, signals at very high *b*-values might be still too much influenced by noise. A thorough evaluation of the signal decay curves is required to select optimal *b*-values for a particular model and anatomical site beforehand, for example by analyzing the goodness of fit when including different *b*-value combinations. An approach of performing such an evaluation is provided in [24] in the context of prostate cancer.

### 4.2 Patient positioning

**Fig. 2:**
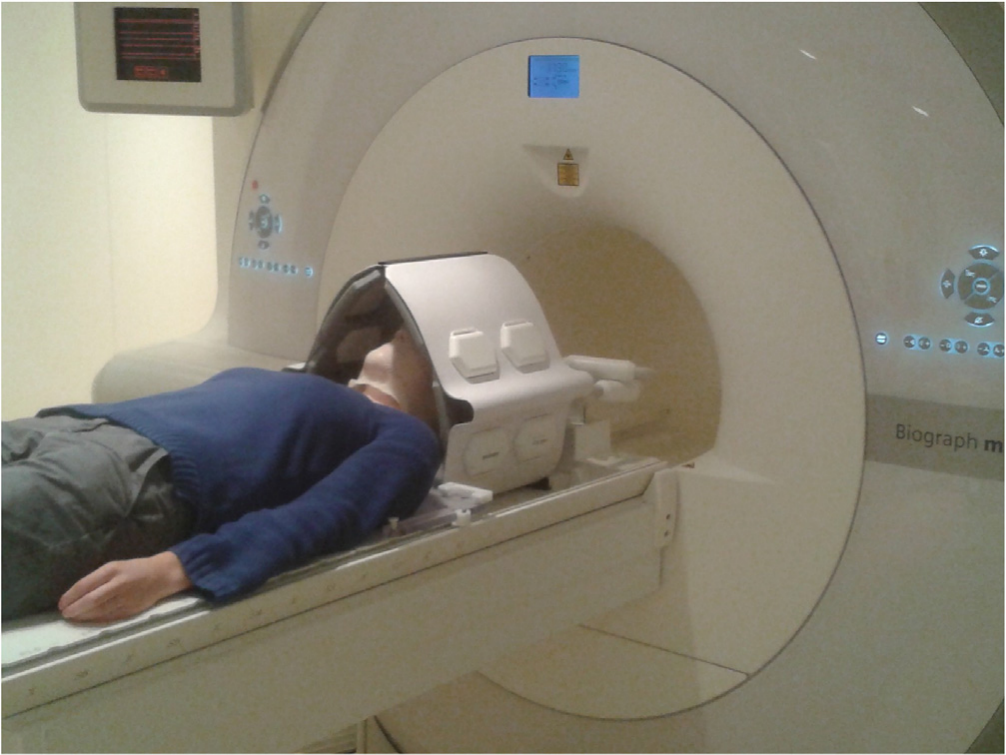
Dedicated positioning solution for MR imaging of head and neck cancer patients in RT treatment position. The components are a flat table top with an add-on for the fixation of a head and neck positioning mask, and coil holders to with flexible RF coils can be attached.

For the integration of DWI into RT planning, imaging should ideally be performed in RT treatment position. As for head and neck cancer patients, positioning solutions generally include a flat table top and a mask fixation system, as well as the usage of flexible RF coils instead of a standard diagnostic head coil to provide enough space for the RT mask [38], [39], [40]. Such a positioning system is shown in Fig. 2. As the signal to noise ratio can be impaired due to the usage and setup of different receiver coils, it is necessary to validate that image quality using RT-setups is sufficient for extracting reliable imaging parameters [41].

## 5 Potential benefits of DWI for RT

There is an increasing number of publications investigating potential benefits of DWI in the context of RT. However, DWI has not found its way into clinical application yet for most cancer sites. Nonetheless, DWI with multiple b-values is recommended as part of minimum requirement for detection, staging and nodal involvement evaluation in prostate cancer by the European Society of Urogenital Radiology (ESUR) [42]. In cervical cancer, DWI has become part of the brachytherapy planning protocol at many institutions, and there is a focus on documenting the added value of DWI for this purpose [43].

In the following, an overview of recent publications about the potential of DWI-derived biomarkers for RT outcome prediction, response assessment, and tumor delineation is provided. A detailed listing of the cited studies is provided in Table 1.

**Table 1:** Previous studies about potentials of integrating DWI in RT. Purpose – 1: pre-treatment outcome prediction, 2: response assessment, inter-treatment prediction, 3: tumor delineation; Mexp - mono-exponential model, IVIM – intra-voxel incoherent motion; NGK – non-gaussian kurtosis, ADC – apparent diffusion coefficient, ΔADC – difference of inter- or post-treatment ADC to baseline; DTI - diffusion tensor imaging, DCE – dynamic-contrast enhanced imaging.

Purpose	Site	Author/year citation	# patients	*b*-Values in s/mm^2^	Imaging time point	Model	Main findings
1	HNSCC	Lambrecht 2014 [44]	161	0, 50, 100, 500, 750, 1000	pre-RT	Mexp (high-, low- and full *b*-value range)	higher pre-treatment ADC in tumor, when derived from the high *b*-value range, is related to disease recurrence
1	“	Noij 2015 [30]	78	0, 750 and 0, 1000	pre-(C) RT	Mexp (ADC_750_, ADC_1000_)	higher pre-treatment ADC_1000_ in lymph nodes is related to lower disease-free survival
1	“	Hauser 2013 [53]	22	0, 50, 100, 150, 200, 250, 700, 800	pre-RT	IVIM	high perfusion fraction *f* in tumor may be related to poor prognosis
1,2	Rectal cancer	Jung 2012 [45]	35	0, 500, 1000	pre- and post-CRT (neoadjuvant)	Mexp	significant correlation between pre-treatment ADC and tumor volume reduction, as well as between ΔADC and tumor volume reduction
1,2	“	Lambrecht 2012 [46]	20	0, 50, 100, 500, 750, 1000	pre-, inter-, and post-CRT (neoadjuvant)	Mexp	pre-treatment ADC as well as inter- and post-treatment ΔADC may be useful for prediction and early assessment of treatment response; pretreatment ADC is significantly lower in patients with pathologic complete response
1	“	Joye 2017 [47]	85	0, 50, 100, 300, 600, 1000	pre-, inter-, and post-CRT	Mexp (high-, low- and full *b*-value range)	DWI is predictive for treatment response; the predictive power can be improved by combining DWI with FDG-PET and T2-weighted volumetry
1	Glioblastoma	Pramanik 2015 [48]	21	0, 1000, 3000	pre-CRT	no model applied	hypercellularity volume as defined on the b = 3000 acquisition is a significant prognostic factor for progression-free survival
1	Cervical cancer	Heo 2013 [49]	42	3 0, 500, 1000	pre-CRT	Mexp	higher mean ADC related to tumor recurrence; 75th percentile ADC predictor for tumor recurrence
1	“	Onal 2016 [50]	44	0, 800	pre-CRT, post-CRT	Mexp	lower ADC values pre-RT and post-RT associated to disease recurrence
1	“	Marconi 2016 [51]	66	0, 600 and 0, 800	pre-CRT	Mexp	Pre-treatment minimum ADC may be a prognostic factor for disease-free survival
1	“	Gladwish 2016 [52]	85	0, 50, 400, 1000, and 0, 100, 800 and 0, 50, 400, 800	pre-CRT	Mexp	95th percentile ADC might be a metric to predict treatment failure
2	HNSCC	Dirix 2009 [56]	15	0, 50, 100, 500, 750, 1000	Pre-, inter-, and post-CRT	Mexp	lesions showing loco-regional recurrence had a significantly lower inter-treatment ADC
2	“	King 2013 [57]	30	0, 100, 200, 300, 400, 500	Pre- and inter-CRT	Mexp	local failure is associated with lower relative increase of ADC compared to local control, as well as with a decrease of skewness and kurtosis in GTV-based ADC histograms
2	“	Marzi 2015 [65]	34	0, 25, 50, 75, 100, 150, 300, 500, 800	Pre-, inter-, and post-CRT	IVIM	pre-treatment *f* and *D* are independent predictors for shrinkage of major salivary glands
2	“	Vandecaveye 2012 [66]	29	0, 50, 100, 500, 750, 1000	Pre- and post-CRT	Mexp	ΔADC three weeks after RT allows for early treatment response assessment
2	Cervical cancer	Haack 2015 [59]	11	0, 150, 600, 1000	Pre- and inter-RT	Mexp	volume with reduced diffusion as derived from DWI changes significantly during treatment, along with a significant mean ADC increase
2	“	Das 2015 [60]	24	0, 400, 800	Pre- and inter-CRT	Mexp	inter-treatment ΔADC can be used for early response prediction
2	“	Zhu 2017 [21]	30	0, 10, 20, 30, 40, 50, 100, 150, 200, 350, 500, 650, 800, 1000	Pre- and inter-CRT	IVIM	*D* at 2 weeks as well as *D* and *f* 4 weeks after start of RT prognostic for therapy outcome
2	“	Daniel 2017 [61]	10	0, 850	Pre-, inter-, and post- CRT	Mexp	Patient averaged ADCs increased from baseline to follow up, low-ADC regions spatially varied over time
2	“	Schreuder 2015 [7]	231 (review)	mixed	Pre-, inter- and post-RT	Mexp	DWI can be used for early post-RT assessment, but not for early response monitoring
2	Glioma	Kassubek 2017 [63]	18	0, 800	Pre- and post-RT	DTI	DTI can potentially be used to asses irradiation-induced microstructural white matter damage
2	Glioblastoma	Nagesh 2008 [64]	25	0, 1000	Pre-, inter- and post-RT	DTI	DTI has potential for the assessment of radiation-induced white matter injury
2	“	Chu 2013 [67]	30	0, 1000 and 0, 3000	post-RT	Mexp (ADC_1000_, ADC_3000_)	Fifth percentiles of cumulative histograms of ADC_1000_ as well as of ADC_3000_ promising for the differentiation between true progression and pseudo-progression
2	Esophageal cancer	van Rossum 2015 [58]	20	0, 200, 800	Pre-, inter-, post-CRT (neoadjuvant)	Mexp	inter-treatment ΔADC is a predictive factor for histopathologic response
3	“	Hou 2013 [68]	42	400, 600, 800	pre-treatment	no model applied	DWI is superior to CT or anatomical MR in GTV delineation
3	Pancreas cancer	Kartalis 2016 [26]	15	0, 50, 150, 200, 300, 600, 1000	pre-treatment	IVIM, Mexp, NGK	ADC and $D_{K}$ might be valuable for differentiating between tumorous and non-tumorous parenchyma
3	Glioblastoma	Jensen 2017 [69]	11	0, 1000	pre-RT	DTI	DTI in combination with a model for the microscopic spread of tumor cells along white matter fiber tracts might be of value for defining the clinical target volume (CTV) of glioblastomas
3	Cervical cancer	Schernberg 2017 [70]	44	0, 1000	after CRT, before image guided adaptive brachytherapy	no model applied	DWI images (without applying quantitative models) might lead to modifications in high-risk clinical target volumes
3	Prostate cancer	Langer 2009 [71]	25	0, 600	pre-treatment	Mexp	ADC is superior to DCE and T2-mapping for differentiating between tumorous and non-tumorous tissue; classification accuracy can be increased by using a multi-parametric model
3	“	Groenendaal 2012 [72]	87	300, 500, 1000	pre-treatment	Mexp	Logistic regression-derived model including DWI, DCE can define different risk levels for tumor presence on a voxel level
3	“	Yu 2017 [73]	140	50, 600, 1000	pre-treatment	Mexp	Multiparametric model of DWI, T1, and T2 may discriminate between tumorous tissue and normal peripheral zone

### 5.1 Pre-treatment prediction of RT outcome

In different tumor sites, a lower mean pre-treatment ADC derived from the gross tumor volume (GTV) has been found to be related to better RT treatment response (cf. Table 1). A possible explanation for this finding might be that tumors with higher mean pre-treatment ADC are likely to be more necrotic [31], and might in consequence also contain more hypoxic areas [44].

As for head and neck squamous cell carcinoma (HNSCC), mean ADC has been identified as a prognostic factor particularly if derived from the high *b*-value range only, whereas mean ADC could not be related to outcome if derived from low *b*-values only [44], [30]. Moreover, several studies show that mean pre-treatment ADC is of predictive value for neoadjuvant radiochemotherapy response in rectal cancer [45], [46], [47] as well as a prognostic factor for progression-free survival in glioblastoma [48]. In cervical cancer, low mean pre-treatment ADC has been associated with good outcome [49], whereas the opposite relationship has also been reported [50]. Other studies found a relation of parameters derived from histograms of ADC distribution within the GTV and treatment response [49], [51], [52]. Perfusion-related parameters have been less studied. However, in HNSCC the lymph node perfusion fraction *f* as derived by IVIM has been related to locoregional recurrence after therapy [53].

### 5.2 Inter-treatment monitoring and outcome prediction, early response assessment

Another application of DWI might be RT monitoring and early response assessment after treatment. Changes in functional MR images often proceed anatomical changes, and might therefore be early indicators of treatment response [15], [54].

Moreover, DWI acquired in the course of fractionated treatment, eventually when set into relation with the acquisition at baseline, might potentially have a greater prognostic value than DWI at baseline alone. A higher increase in ADC during therapy seems to be related with a better outcome for the patient (cf. Table 1), which might likely to be attributed to reduced cellularity in the tumor caused by treatment-induced cell death [44], [55].

DWI has been shown to be of value in monitoring tumor changes as well as in predicting outcome during fractionated RT in different cancer types, such as the HNSCC [56], [57], esophageal cancer [58] and cervical cancer [59], [60], [21], [61]. In the treatment of cerebral tumors, microstructural damage of white matter may be detected by directional diffusion anisotropy derived from DTI [62], [63], [64]. In addition to tumor monitoring, DWI might also be used to monitor organs at risk during treatment, such as the major salivary glands in head and neck RT [65]. With respect to the assessment of early treatment response after therapy in terms of locoregional failure or metastasis detection, DWI has been found to be promising in HNSCC [66], glioblastoma [67], cervical cancer [7], and rectal cancer [46].

### 5.3 Tumor delineation, local dose escalation

Voxel-based DWI analysis might be of value for tumor delineation, e.g. in esophageal tumors [68], pancreatic cancer [26], as well as for glioblastoma [69]. In cervical cancer, DWI might have an impact on the definition of high-risk clinical target volumes [70]. In prostate cancer DWI has been reported to be a promising tool for the differentiation of tumor from non-tumor tissue, especially when combined with other functional imaging modalities [71], [72], [73]. Based on [72], a phase III multicenter trial (FLAME) is currently investigating the effects of a local dose boost to the macroscopic prostate tumor as derived from functional MRI including DWI [74], [75], [76]. However, to the best of our knowledge, investigations for using DWI with respect to intra-tumor treatment adaptation have been limited to planning studies so far. A more detailed discussion of potential strategies for such an adaptation is given in Section 7.

## 6 Challenges with respect to DWI accuracy

### 6.1 Geometric distortions

As DWI is very sensitive to motion-induced phase errors, it is usually acquired using rapid echo-planar imaging (EPI) sequences [77]. One drawback of EPI sequences is that they are prone to geometric distortions, which are most prominent in phase-encoding direction. They are induced by inhomogeneities of the static magnetic field $B_{0}$, which can arise from external inhomogeneities or induced internally by susceptibility differences within the imaged object or patient. In addition to geometric distortions, $B_{0}$ inhomogeneities are also accompanied by signal loss, which leads to lower quantification accuracy. Adverse effects are most prominent in regions with strong susceptibility changes such as the head and neck [78], [79], [80].

For the integration of DWI into RT treatment planning, high geometric and quantitative accuracy is crucial. Thus, it is important to address this problem by preferably using an EPI sequence which is optimized towards lower distortions.

One way to address this problem is to choose acquisition parameters in favor of low distortions, such as a high receiver bandwidth and parallel imaging [80]. One possibility to further reduce distortions is the usage of readout-segmented multishot EPI sequences, which have shown to greatly reduce geometric distortions by shortening effective echo time [81], [82], [83]. Another option is to combine EPI acquisitions with integrated dynamic shimming, in which the off-resonance field $\Delta B_{0}$ is reduced by adjusting shim and excitation frequency slice-per-slice [84]. Combining these two techniques is promising to further increase geometric accuracy; however, at the expense of a longer imaging time [85].

The above-mentioned sequences (readout-segmented EPI and integrated shimming) require the availability of dedicated sequences from the vendor. If no such implementation is available, it is possible to acquire an additional image with reversed phase encoding direction (RPED) for each *b*-value, and to calculate an undistorted image by a registration-based approach [40], [86]. However, this technique relies on a post-processing step and cannot compensate for signal loss.

Another alternative is to use a reduced field of view (rFOV) technique applying 2D spatially selective excitation pulses, exciting only a small inner volume along the phase-encoding direction. This technique allows a reduced number of phase-encoding steps, leading to higher resolution imaging for a fixed scan time as well as reduced geometrical distortion [87]. rFOV-DWI been successfully applied to imaging of the prostate, spinal cord, breast, rectum, and uterine cervix [87], [88].

Furthermore, inherently less distorted non-EPI secquences may be used, such as periodically rotated overlapping parallel lines with enhanced reconstruction (PROPELLER) [89], [90]. Up to now, only few studies evaluating these sequences with respect to DW image quality exist. The advantages and drawbacks in terms of imaging time, image artifacts and signal-to-noise ratio have to be considered along with the extent of geometrical distortions.

### 6.2 Accuracy of DWI-derived parameters

Apart from geometric accuracy, robust quantification has to be addressed [91]. For robust derivation of quantitative parameters from DWI, the uncertainty in the acquired signals in terms of noise and artifacts should be within reasonable limits for the applied model. This issue is especially critical if voxel-based parameter maps are to be derived, as uncertainty of measured samples on a voxel-wise basis is much higher compared to averaged regions. As the fit quality is usually not given in vendor-provided parameter maps, it might be valuable to take a closer look at the originally acquired data. In the ideal case, in–house fits should be performed to allow for quantifying the accuracy of derived parameters, for example by exploiting information of the fit covariance matrix. Especially when using complex models such as IVIM or if voxel-based parameter maps should be derived, high-quality data with multiple *b*-values as well as a thorough evaluation of fit quality are necessary [19].

## 7 Discussion

Different DWI-derived parameters such as ADC assessed from the mono-exponential model, and IVIM model parameters have been investigated as imaging biomarkers for RT, with promising results for outcome prediction, therapy monitoring and early response assessment (cf. Table 1). Up to now, mono-exponential models have been applied in most studies, whereas the IVIM and Kurtosis models have been investigated in only few studies.

A general drawback of the current research is that the studies often have small sample sizes and comparability between studies is limited due to the use of different DWI protocols and evaluation strategies. No standardization for DWI protocols and evaluation is available yet, but potential guidelines are being discussed [92].It is however reasonable that the choice of imaging parameters should depend on the purpose of the study and on the model which is to be applied for data evaluation. It is highly important to address both geometric accuracy and robustness of quantitative parameters when designing a DWI study, especially if parameters should be obtained on a voxel-wise basis, or if complex models such as IVIM should be used for data analysis.

In most studies, single parameters from functional imaging have been investigated for their potential use as biomarkers in RT. In addition to DWI parameters, other MR-based parameters derived from dynamic contrast-enhanced (DCE) MR imaging, MR arterial spin labeling (ASL), and MR spectroscopy have been studied [3], [93], [94]. Also PET imaging using tracers such as [^18^F]-fluoromisonidazole (FMISO) or FDG might be highly beneficial the context of RT [95], [96], [97]. Which imaging and parameter extraction method provides the most benefit for RT is a matter of ongoing research. A combination of several functional parameters might outperform the usage of single parameters. However, such multi-parametric approaches have been investigated only in few studies [5], [71], [72], [73], [98].

Most studies have been focusing on evaluating mean DWI parameters of the GTV. The potential of DWI parameter maps evaluated on a voxel-by-voxel basis has not be thoroughly studied yet. For example, histograms showing the distribution of DWI parameters within the GTV could potentially contain more accurate predictors for RT outcome than mere GTV-averaged parameters alone [57]. Moreover, voxel-by-voxel parameter maps may help in differentiating between cancerous and non-cancerous tissue, and therefore support tumor delineation [74], [75], [76]. Local biological characterization of the tumor could additionally support intra-tumor dose escalation strategies [3], [4], [5].

To the best of our knowledge, except for the FLAME trial (cf. Section 5), clinical studies for treatment adaptation based on DWI parameters have not yet been performed. Different concepts for adapting therapy to functional image information within the tumor are available such as a boost of RT dose to the whole tumor volume, region-based adaptation by the definition of boost doses to biological target volumes, or a voxel-based adaptation of dose such as dose-painting by numbers (DPBN) [99]. First planning studies for intra-tumor RT dose adaptation based on DWI have demonstrated the dosimetric feasibility [100], [101]. Due to the predictive value of DWI for treatment outcome dose adaptations seem promising, however, comprehensive clinical studies are required to prove the validity of this concept. Another potential approach to validate DWI-based biomarkers might be the investigation of correlations to more established biomarkers such as FMISO or FDG, for which already studies with promising results with respect to treatment adaptation are available [102].

## 8 Conclusion

DWI has potential to improve RT, and may be used for outcome prediction, early response assessment, as well as tumor delineation and characterization. Challenges such as geometric and quantification robustness need to be addressed adequately. Further research with respect to deriving biomarkers and how to implement them into RT appears promising.

## Conflict of interest statement

The authors declare that they have no conflict of interest.
